# Monthly Summary

**Published:** 1867-09

**Authors:** 


### MONTHLY SUMMARY.

*Atropine harmless to Rabbits.*—Dr. Ogle, Lecturer on Physiology to St. George's Hospital, publishes in the "*Medical Times and Gazette,*" a paper on the "Comparative Immunity of Rabbits to the poisonous action of Atropine."

It has been known for many years that rabbits could eat the leaves of belladonna without inconvenience and Runge showed that this was not for want of power to absorb the poisonous principle, since he detected the presence of atropine, the active alkaloid, in the animal's urine. Dr. Ogle took up first the question; can rabbits eat belladonna with impunity? He fed a healthy young rabbit for six days exclusively on this plant, having first proved the activity of the herb by applying crushed leaves to the eye of a kitten and ascertaining that the pupil was dilated by it. The health of the animal was not impaired.

The next question to answer was: Is atropine absorbed by rabbits, and, if so, is it as poisonous to rabbits as it is to man? Men are easily killed by two grains of atropine administered by the mouth, and are seriously injured by from 1-10th to 1-20th of a grain, hypodermically injected. Experimenting on rabbits, it was found that two grains administered by the mouth produced no inconvenience, while so much as five grains injected under the skin gave only slight and temporary uneasiness. The absorbtion of the poison was proved by applying some of the rabbit's urine to the eye of a cat, when strong dilatation of the pupil occurred. Dr. Ogle did not push his inquiries to the extent of inquiring what quantity of atropine would kill a rabbit, that having already been determined by Dr. Camus, who found the mininum quantity necessary for that purpose to be about fifteen and a half grains.

It having been suggested that young rabbits might enjoy an immunity not to be found in older animals, experiments were made to determine this question. It was discovered that youth established no such immunity, that on the contrary, the tolerance increased with the age of the rabbit, while the single symptom of dilatation of the pupil was as readily induced in the old as in the young rabbit.

---

*Cyst in the Lower Jaw.*—At King's College Hospital, there was a case of tumour in the lower jaw, caused by expansion of its walls. Sir W. Fergusson extracted a tooth and then broke into the cavity, removing the contents which consisted of a small quantity of gelatinous material. He had seen similar cases in which the walls of the antrum expanded in consequence of the formation of such semifluid growths in a bony cyst, growths which had been entirely cured by puncture and evacuation of the contents. In one instance he removed a large portion of the lower jaw, and found between the expanded flexible laminæ of the body of the jaw, a large mass of dentine. In this case he thought the cavity should have been scooped out before removal was attempted.

---

*New Discovery in Comparative Anatomy.*—It is known to our readers that a division of mammalia has been established on the history of their dentition. Thus some, the Cetacea and Bruta, never shed their first teeth, while the rest go through the same changes as man. Thus we have Monophyodonts, or animals which have but one set of teeth, and Diphyodonts, or animals which have two sets.

Now Mr. Flower, the conservator of the Museum of the Royal College of Surgeons, tells us that the Marsupial, or pouched animals, such as the opossum and kangaroo, stand intermediate between these two great classes, for in them is traceable the first step towards a double set of teeth, the representative of the deciduous set being confined to a single tooth, a milk molar, which is succeeded by the anterior premolar on each side in both jaws. None of the other permanent teeth have any predecessors, though perhaps some trace of their follicular stage might be found at a very early period of their development.

This is not unlikely, since it is known that in some instances, for example, in a few Rodentia, the milk teeth are shed *in utero*. This opinion, however, is merely hypothetical; actual observation never having detected any traces of deciduous teeth in Marsupials. It is a little remarkable that the deciduous tooth alone developed in these tribes of animals, is usually the most persistent in typical Diphyodonts and in man, viz: the posterior milk molar replaced by the posterior premolar.

---

*Deodorization of Caoutchouc.*—M. Bourne encloses articles of India rubber in a vessel, surrounding them with charcoal dust, and heats them to 60° or 70° C. for several hours. The charcoal after cooling is removed, and the articles are found unchanged in their minutest details, but completely deodorized. India rubber thus prepared communicates no unpleasant taste to food. Either hot water, steam, or the heat of the vulcanizer may be employed to raise the temperature to the proper standard.

We get the above from the foreign correspondence of the *London Chemical News.*

---

*The Human Bite Poisonous.*—A singular occurrence has just happened at Arth, in France. A Lieutenant Felchin was some time back bitten in the thumb by a man named Muller, but he thought nothing of the wound and went next day a journey on his private affairs. On reaching the Balse he found his hand and arm began to swell, and a medical man declared that the case was one of poisoning from a human bite. He at once returned home in haste, but he refused to have the arm amputated, The consequence was that the inflammation increased frightfully, and he died some days after in horrible suffering. May not the system have been at fault?

---

*Quinine as a preventive of Malarial disease.*—Dr. Joseph Jone has published in the *Nashville Journal of Medicine and Surgery,* a paper on the above subject.

Statistics of British ships on the coast of Africa collected by Dr. Jones very strikingly illustrate the value of this prophylactic treatment. For example, of 145 whites on the Niger expedition, 130 were attacked with fever, and 142 died, and of 525 soldiers garrisoning Sienna Leone, 386 were attacked and 161 died. These are specimens of the statistics of commands in which quinine was not administered.

On the other hand, in the African squadron, cruising off this same coast in 1856, to the officers and men of which quinine was daily administered, out of a total force of 1680 men, only seven deaths from fever occurred.

Dr Jones lays down the following propositions:

"1. Quinine taken during exposure to the exhalations of miasmatic regions will, in most cases ward off fever entirely.

2. If fever attacks those to whom quinine has been regularly administered, its severity and duration will be much less than in those who have not taken the quinine; it therefore not merely wards off disease but renders it less powerful and destructive when present.

3. To be entirely efficient, the quinine must be administered for some time, at least ten days affer exposure to the causes of fever."

The formula recommended by Dr. Jones is:

Sulphate of Quinine.................. ........................3 grains
Dilute aromatic Sulphuric acid..........................5 drops
Brandy.............................................Tablespoonful
Water.................................... .......3 wine glassfulls

Drop the acid on the quinine, then add the brandy and water. Take the whole just after rising in the morning, and again just before going to bed at night.

---

*Local Anaesthesia by cold.*—Dr. JAMES ARNOTT says (*Med. Times and Gaz.*) "nothing can be more easy than to dip a bit of ice into common salt and press it gently on the skin, and yet this is sufficient to freeze it in less than the quarter of a minute. In some cases, indeed, a frigorific mixture cannot be properly applied without a cup or vessel of peculiar shape to contain it, which unquestionably involves more trouble than the projection of ether; but in several situations the part cannot be congealed by ether, and it is necessary to use either a freezing mixture, or a metallic ball or oval which has been cooled to the requisite degree by immersion in it. When deep, extensive, and long-continued congelation is required, or when it has to be used when inflammation is present, either in operations or in the treatment of disease, congelations by a freezing mixture, which can be combined with pressure and applied to any extent of surface, is the only measure which will fulfill the purpose. Simplicity and facility of application are doubtless valuable properties, but efficiency must not be sacrificed to ease."

---

"*Sloughing produced by Local Anaesthesia.*—We examined, a few days since, in the Middlesex Hospital, a young woman whose case is of no little importance in reference to the question of local as against general anæsthesia for operations. Mr. Lawson had diagnosticated the existence of an abscess behind the patient's breast, and as the pus was very deep (under the pectoral muscle indeed) the refrigerator was used, paraffine ether being employed. Congelation was rapidly produced, and kept up for a few minutes. The result has been, that a portion of skin, about an inch by three-quarters of an inch, over the upper part of the breast, has sloughed, and its healing will necessarily be attended by an unseemly scar. The patient is a maid-servant; were she unfortunately a lady, the undress of the modern ball-room would be impracticable without revealing such a blemish as might seriously damage her value in the matrimonial market. The case is certainly exceptional; but the circumstance is worth remembering when exposed parts of the body are to be operated upon." —(*Lancet.*)

---

*Marine Silk.*—Among the novelties brought out in the great Paris Exhibition is a silk originating in the sea. M. Joly has discovered that the outer envelope of the eggs of the Ray tribe of fishes is made up of very delicate filaments closely interwoven. They are easily drawn out, and possess the colour and finish of woven silk, and can be woven into all ordinary silk tissues. The interior of the egg contains an albumen which can substitute, in the arts, that heretofore derived from hen's eggs.

---

*Making Light of the Dead.*—An ingenious correspondent of the *Gazette Medicale de Lyons*, alarmed at the rapid diminution of the coal supply, proposes to utilize dead human bodies, which are now wasted. He estimates that an average sized corpse will yield 25 cubic metres of illuminating gas, so that any ordinary body would be worth for this purpose eight francs.

---

*A New Parasite.*—Dr. Judee, of Bizot, describes a new parasite which he recently found principally about the neck, the breast, and the hair of women The parasite in size and aspect resembles a small dark speck the size of a pin's point. With the naked eye little dark lines are seen in its hard body, and in consequence of the smallness of the object, are confused together. Examined with a power of 140, the following elements are noticed :— A head furnished with two antennæ, an abdomen and four pairs of legs, each composed of four articulations, and armed at its extremity by a species of claw. The body and legs are covered with hair. The three dark lines are not superficial, but deep, and depend upon the organs contained in the abomen. It is very lively, moving rapidly about; and it eats its way, leaving behind a little black powder of extreme fineness. Once separated from the body, it quickly dies. Dr. Judee observes that he has found this parasite on individuals who wear false hair ; but he does not think it has any necessary connexion with it. It is common, perhaps, in the Arabs during Rhamadan. The treatment practised was antiparasitic. The insect appears to be a tick.—*Lancet.*

---

*French Malleable Horn.*—A French scientific journal gives an account of a new process for making malleable horn. The horn, in chips and shavings, is boiled a long time in a caustic lye of strength of twenty-five degrees of the alkalimeter, by which it is entirely melted. The liquid is then reduced by evaporation to a plastic paste, which may be rolled into sheets, drawn into rods, or moulded in any form. This paste is rendered more strong and elastic by mixing it with india-rubber or gutta-percha. The substances are mixed together in a cast-iron vessel, and passed between fluted revolving rollers, the vessel being heated by steam. The inventors state that, by covering the fibres of cocoa or of aloes with this paste, they have obtained belts more solid than those of leather, and stronger than those of india-rubber.

